# Continuing Care for Mentally Stable Psychiatric Patients in Primary Care: Patients' Preferences and Views

**DOI:** 10.1155/2012/575381

**Published:** 2012-07-11

**Authors:** Vincent I. O. Agyapong

**Affiliations:** ^1^Department of Psychiatry, The Trinity College, University of Dublin, Dublin, Ireland; ^2^Department of Psychiatry, St. Patrick's University Hospital, James Street, Dublin 8, Ireland

## Abstract

*Objective*. To investigate the preferences of psychiatric patients regarding attendance for their continuing mental health care once stable from a primary care setting as opposed to a specialized psychiatric service setting. *Methods*. 150 consecutive psychiatric patients attending outpatient review in a community mental health centre in Dublin were approached and asked to complete a semistructured questionnaire designed to assess the objectives of the study. *Results*. 145 patients completed the questionnaire giving a response rate of 97%. Ninety-eight patients (68%) preferred attending a specialized psychiatry service even when stabilised on their treatment. The common reason given by patients in this category was fear of substandard quality of psychiatric care from their general practitioners (GPs) (67 patients, 68.4%). Twenty-nine patients (20%) preferred to attend their GP for continuing mental health care. The reasons given by these patients included confidence in GPs, providing same level of care as psychiatrist for mental illness (18 patients or 62%), and the advantage of managing both mental and physical health by GPs (13 patients, 45%). *Conclusion*. Most patients who attend specialised psychiatric services preferred to continue attending specialized psychiatric services even if they become mentally stable than primary care, with most reasons revolving around fears of inadequate psychiatric care from GPs.

## 1. Introduction

In Ireland community mental health care consultations are provided by both primary care and specialised public mental health services. However, unlike the specialised public mental health service consultations, primary care service consultations are not currently free for the majority of Irish residents. Patients on the lowest end of the socioeconomic ladder qualify for state “medical or GP visit cards” which are a form of state health insurance that entitles the holder to receive free primary care consultations in addition to the universally free public specialist mental health consultations. All other patients including those with private health insurance pay for all primary care consultations which average around 60 dollars per consultation. A detailed description of the structure of both primary care and community mental health service provision in Ireland, as well as the proportions of patients with medical/GP visit cards and private health insurance and the packages received by these patients have been given in two related publications [1, 2].

The delivery of mental health services presents several important challenges to health systems [3]. The Irish Department of Health Statement of Strategy 2008–2010 emphasizes that the proportion of care needs met in community should be maximized with a shift from hospital system to primary care setting [4]. This is consistent with international studies which have reported that patients with mental health difficulties reported no deterioration in their clinical condition while under the care of general practitioners (GPs). It is also consistent with reports that formal liaison between GPs and specialist services improves functional outcomes in chronically mentally ill patients [5, 6]. In line with these observations, one study reported that patients with mild to moderate depression and anxiety found it more acceptable to receive psychiatric care from primary than from specialized psychiatric services [7]. 

In 2003, the Government of Ireland commissioned an expert group to review Ireland's mental health policy. The policy document produced by this expert group in 2006, entitled: “A Vision for Change,” recommends that links between specialist mental health services, primary care services, and voluntary groups that are supportive of mental health should be enhanced and formalised [8].

 However, some studies have identified challenges including patients' lack of confidence in GP care, GPs not being adequately trained to provide mental health care, and primary care not being adequately resourced with a multidisciplinary team to facilitate the care of patients with mental health difficulties, which cause some service users deemed to have relatively low clinical needs to remain within the specialist psychiatric service [1, 2, 9, 10]. A survey of a section of GPs working in Ireland in 2003 indicated that despite the fact that 35% of general practice attendees have mental health problems, and that over 95% of these problems are dealt with in primary care, only 32% of GPs had received postgraduate training in psychological therapies [11]. There is also evidence that suggests that GPs are willing to take responsibility for physical health care [10, 12] but do not perceive themselves as involved in the mental health or overall care of people with enduring mental illness [10, 13].

This study explores the preferences and views of patients attending a local community mental health clinic regarding their continuing care with primary care or specialist mental health services once they have been stabilised on their treatment.

## 2. Methods

As no prior questionnaire existed, a self-administered questionnaire of 20 items based on literature review and reflecting the objectives of our study was designed by the researcher. The questionnaire included questions on patients demographic and clinical characteristics such as age, sex, marital and employment status, possession of state or private medical insurance, frequency of attendance of psychiatric clinic, presence of medical comorbidity, registration with and frequency of attendance of general practice surgery, preference for receiving continuing care from primary care or specialised psychiatric services when mentally stable on treatment, and reasons for the choice. It was reviewed by a multidisciplinary group of six experts (two researcher and four psychiatrists) and pretested with five patients who were not included in the study sample. It took approximately 10 minutes to complete, and no financial incentive was offered to respondents. 150 consecutive psychiatric patients attending for outpatient review in a community mental health centre in Dublin were approached and asked to complete the questionnaire. The study received approval from the Research and Ethics Committee of the College of Psychiatry of Ireland.

After complete description of the study to the participants, written informed consent was obtained before they completed the survey forms. This cross-sectional survey was conducted between April and June 2009 and data were analysed using descriptive statistics and Chi-square test with SPSS version 17.

## 3. Results

145 patients completed the questionnaire giving a response rate of 97%. The mean age for all respondents was 45.48 year (SD = 13.01 years) and the mean years for which patients had been attending the local specialised psychiatric service was 8.6 years (SD = 8.1 years).  Table 1 gives the other demographic and clinical characteristics of the respondents. 

Overall, 42 (29%) patients reported that they had been stable on their medication for more than one year while 13 (9%) patients reported that they had been stable on their medication for between 6 months and one year. Again, 39 (27%) patients had had no changes made to their psychiatric medicines in over one year, 16 (11%) patients were on a depot injection, and 36 (25%) patients had a community psychiatric nurse assigned to them for follow-up visits. 45 (31%) patients reported that on the average, they attend the psychiatric clinic for review once every month, 48 (33%) said they attend about once every six weeks, 34 (23%) reported that they attend once every two months, and the rest said they attend once every 3 or more months. Within the previous three months, 71 (49%) patients reported that they have visited their GP between one and three times and 19 (13%) reported they had visited their GP more than three times for a medical review. Overall, 32 (22%) patients reported that they attended their GP regularly for review and prescriptions for chronic medical conditions. Of the 83 patients who have had previous inpatient psychiatric treatment, 57 (69%) reported that their inpatient treatment had been more than a year previously and the remainder had been within the last one year.

## 4. Patients' Preferences Regarding Continuing Care

Overall, 29 (20%) patients indicated that they would prefer to attend their GP for their psychiatric care once they have been stabilised on their medication from specialised psychiatric services. 98 (68%) patients expressed a preference to continue attending specialised psychiatric services even if they are stable on their treatment and the remaining 18 (12%) patients expressed no preference for either primary care or specialised psychiatric services. I performed a Chi-square test to examine the association between the demographic and clinical variables (including gender, age group, relationship and employment status, possession of a medical card or private health insurance, and the presence of a medical comorbidity) and the expression of a preference to receive continuing care from primary care. I found no significant association between any of these demographic and clinical variables and the expression of a willingness to receive continuing psychiatric care from primary care (*P* > 0.05 for all comparisons).

The reasons given by patients who stated a preference for and against attending their GP for their psychiatric needs once they are stable on medication are as outlined in Tables 2 and 3, respectively.

## 5. Discussion

This study has established that the majority of Irish patients (98, 68%) would prefer to continue receiving mental health care from specialised psychiatric services rather than receive such care from primary care even if they become stable on their treatment. The majority of these patients (67, 68%) reported that they would be worried about the quality of the psychiatric care they would receive from their GP even if they are stable on their treatment. Many of these patients (40, 41%) also expressed that they did not have a medical or GP visit card and cannot afford to pay for GP consultations. In one multidisciplinary team's retrospective review of the clinical notes of all patients attending a psychiatric outpatient clinic to determine the appropriateness of continuing to provide psychiatric services in a specialised mental health clinic rather than in a primary care setting, it was recommended that 35.2% of all the patients could be discharged back into primary care for continuing management [14]. Community mental health services usually come under financial pressure and there are significant constraints hampering the effective management of the volume of consumers under their care, leading usually to long waiting times for access to psychiatric services. These tend to cause concerns among GPs and the community generally, including confusion and bewilderment in GPs when faced with difficult mental health problems for which they could not easily access support from specialist care [3, 15]. Findings from my study indicate that the two main challenges hampering the transfer of care of stable mental health patients into primary care are the lack of confidence in GP care and the cost of GP consultations relative to the relatively free consultations from specialised psychiatric services. Without addressing these two challenges, specialised psychiatric services would have to cope with caring for relatively stable patients which tend to diminish their capacity to take on new referrals from primary care.

Of the 29 (20%) patients who reported that they would prefer to attend primary care for their continuing mental health care once they are stable on their treatment, 18 (62%) stated that they had confidence in the care they would receive from their GP whilst 13 (45%) said it would be handy for them to attend their GP for both their physical and mental health needs. Again, 9 (31%) of these patients said that receiving continuing mental health care from primary care would take away the stigma often attached to attending psychiatric services.

Providing good generalist care which addresses both mental and physical health needs is recognised internationally as one of the benefits of primary care service involvement in the care of patients with mental health difficulties [16].

This study suggests that if a similar set of conditions exist in terms of the cost of consultations between primary care and specialised psychiatric services, and if GPs were adequately trained to provide continuing care for patients with enduring mental health difficulties, then the capacity of specialized psychiatric services to take on new referrals as well as care for acutely unwell patients would be greatly enhanced. Two previous surveys of GPs working in Ireland indicate that those GPs who undertook a psychiatric rotation as part of their GP training expressed significantly more confidence in their ability to recognise and manage psychiatric presentations in primary care compared with GPs who did not undertake such training [2, 11]. To achieve optimal outcomes for people with common mental disorders discharged back into primary care, it is essential that primary care teams have training in good practice guidelines on assessment, diagnosis, management, criteria for referral, and methods of shared care if necessary [17]. Addressing deficits in the knowledge and skills base of GPs in the management of mental health conditions should be coupled with changes in the policy that makes it financially burdensome for patients to receive mental health care from GPs when the same services are provided free of charge in public mental health clinics.

The results of the study should be considered within the context of a number of limitations. Firstly, this study does not take into account the views of psychiatrists and GP's which are described elsewhere [1, 2]. Secondly, it does not take into account the views of mental health patients who are cared for solely in primary care, thus creating a source of bias for our results. Thirdly, although the questionnaire used was reviewed by a multidisciplinary team, it was nonetheless not standardised and so, the results may not be generalisable to other jurisdictions. Furthermore, the multidisciplinary team that reviewed the questionnaire used in the study did not include a GP which is an important drawback. Again, the patients sampled were selected from only one community psychiatric catchment area in Dublin and so, the responses may not be nationally representative. Notwithstanding these limitations, the results of this study are hugely important as they underscore the need to promote collaborative strategies and policies in mental health care.

## 6. Conclusion

Collaboration between GPs and community mental health teams can improve and expand access to care for people with mental illnesses. Most patients who attend specialised psychiatric services in Ireland prefer to continue attending specialized psychiatric services even when they are mentally stable on their treatment as opposed to attending primary care. The most reasons for this revolve around fears of inadequate psychiatric care from GPs and the cost of GP consultations relative to the free consultations they get from specialised psychiatric services. This can significantly restrict the capacity of specialised community mental health teams to take on new referrals from primary care. The Irish Government, the College of General Practitioners, and the College of Psychiatry of Ireland need to work collaboratively to remove the bottlenecks which hinder the active involvement of primary care in the management of patients with enduring mental health difficulties.

## Figures and Tables

**Table 1:** Demographic and clinical characteristics of study population.

Variables	Total *N* = 145 *n* (%)
Gender	
Male	75 (52%)
Female	70 (48%)
Age group	
≤45	75 (52%)
≥46	70 (48%)
Marital status	
Single/divorced/separated/widowed	36 (25%)
Married/cohabiting	109 (75%)
Formal educational background	
Primary	48 (33%)
Secondary	62 (43%)
Tertiary	35 (24%)
Employment Status	
Employed	45 (31%)
Unemployed/retired	100 (69%)
Registered with a GP practice	
Yes	136 (94%)
No	9 (6%)
Has a medical card	
Yes	84 (58%)
No	61 (42%)
Has private health insurance	
Yes	26 (18%)
No	119 (82%)
Diagnosis patients' have been informed they have	
by psychiatrist^∗^	
Depressive disorder	81 (56%)
Bipolar affective disorder	22 (15%)
Anxiety disorder	61 (42%)
Schizophrenia/schizoaffective disorder	16 (11%)
Alcohol dependency syndrome	9 (6%)
Drug/polysubstance abuse	6 (4%)
Other disorder	16 (11%)
Not been told of diagnosis	22 (15%)
Previous psychiatric inpatient treatment	
Yes	83 (57%)
No	62 (43%)
Medical comorbidity	
Yes	41 (28%)
No	104 (72%)

*Some patients have been told they have more than one diagnosis (comorbid disorders).

**Table 2:** Reasons why some patients would prefer to attend their GP for their continuing psychiatric care.

Reason	^ ∗^Total *N* = 29 *n* (% )
I am confident my GP can provide me with the same level of care I would receive from here	18 (62%)
It would be handy for me to attend my GP for both my physical and mental health problems	13 (45%)
It would take away the stigma often attached to attending psychiatric services	9 (31%)
I have a medical card and would attend my GP free of charge	5 (17%)
Other reasons	4 (14%)

^∗^Total *N* is the number of patients who expressed a preference for attending their GP for their continuing mental health care once they are stable on their treatment.

**Table 3:** Reasons why some patients would prefer not to attend their GP for their continuing psychiatric care.

Reason	^ ∗^Total *N* = 98 *n* (% )
I would be worried about the quality of the psychiatric care from my GP	67 (68%)
I do not have a medical/GP visit card and cannot pay for GP consultations	40 (41%)
I do not have a GP	9 (9%)
Other reasons	19 (19%)

^∗^Total *N* is the number of patients who expressed a preference for attending specialised psychiatric services for their continuing mental health care even if they are stable on their treatment.
